# Evaluation of phylogenetic footprint discovery for predicting bacterial cis-regulatory elements and revealing their evolution

**DOI:** 10.1186/1471-2105-9-37

**Published:** 2008-01-23

**Authors:** Rekin's Janky, Jacques van Helden

**Affiliations:** 1 Laboratoire de Bioinformatique des Génomes et des Réseaux, Université Libre de Bruxelles (ULB), Campus Plaine, CP 263, Boulevard du Triomphe, 1050 Bruxelles, Belgium

## Abstract

**Background:**

The detection of conserved motifs in promoters of orthologous genes (phylogenetic footprints) has become a common strategy to predict cis-acting regulatory elements. Several software tools are routinely used to raise hypotheses about regulation. However, these tools are generally used as black boxes, with default parameters. A systematic evaluation of optimal parameters for a footprint discovery strategy can bring a sizeable improvement to the predictions.

**Results:**

We evaluate the performances of a footprint discovery approach based on the detection of over-represented spaced motifs. This method is particularly suitable for (but not restricted to) Bacteria, since such motifs are typically bound by factors containing a Helix-Turn-Helix domain. We evaluated footprint discovery in 368 *Escherichia coli K12 *genes with annotated sites, under 40 different combinations of parameters (taxonomical level, background model, organism-specific filtering, operon inference). Motifs are assessed both at the levels of correctness and significance. We further report a detailed analysis of 181 bacterial orthologs of the LexA repressor. Distinct motifs are detected at various taxonomical levels, including the 7 previously characterized taxon-specific motifs. In addition, we highlight a significantly stronger conservation of half-motifs in Actinobacteria, relative to Firmicutes, suggesting an intermediate state in specificity switching between the two Gram-positive phyla, and thereby revealing the on-going evolution of LexA auto-regulation.

**Conclusion:**

The footprint discovery method proposed here shows excellent results with *E. coli *and can readily be extended to predict cis-acting regulatory signals and propose testable hypotheses in bacterial genomes for which nothing is known about regulation.

## Background

A major challenge of current genomics is to decipher the regulation of the expression for all the genes of a genome. Transcriptional regulation is mediated by interactions between transcription factors (TF) and specific cis-acting elements. The identification of putative transcription factor binding sites (TFBS) is far from trivial, given their short size (typically 6 to 25 bp), and the low level of information in the signal (typically restricted to 5–10 informative nucleotides). For this reason, specific algorithms have been developed to detect cis-acting elements in non-coding sequences. Several pattern discovery algorithms were developed to discover putative regulatory motifs in sets of co-regulated genes from the same genome [1-11].

The ever-increasing number of sequenced genomes opens the possibility to apply comparative genomics in order to analyze taxonomical conservation and divergence between cis-acting elements. This is particularly true for Bacteria, for which the NCBI distributes 548 complete genomes and reports 840 on-going sequencing projects (October 2007). The approach called *phylogenetic footprinting *relies on the hypothesis that, due to selective pressure, regulatory elements tend to evolve at a slower rate than surrounding non-coding sequences. This approach has been applied to detect conserved elements in the regulatory regions of selected mammalian genes, such as globins, *rbc *genes, TnF-alpha [12-16], and in completely sequenced bacterial genomes [17-19] or fungal genomes [20].

A first essential parameter is the choice of the algorithm. Kellis and co-workers [20] used ClustalW, a multiple global alignment algorithm [21]. PhyME [22], EMnEM [23] and orthoMEME [24] are based on Expectation-Maximization, PhyloCon uses a greedy algorithm [25], PhyloGibbs combines *DiAlign *for local alignment and *gibbs *sampling for motif discovery [26]. Another crucial choice is the taxonomical level at which the analysis is led. The initial articles were restricted to the few genomes available at that time, and encompassed various levels of taxonomies: 4 or 5 genomes of the genus *Saccharomyces *[20,26], 9 genomes in the class Gamma-proteobacteria [18], or 17 genomes distributed over the whole bacterial kingdom [17].

With larger numbers of genomes, one can even address the problem in a more general way by comparing motifs discovered at various taxonomical levels, in order to detect not only conservation but also divergence between regulatory elements. This functionality is offered by *Footprinter *[27], which takes as input a set of upstream sequences and a corresponding taxonomic tree, and searches for conserved elements in each branch of the tree.

The choice of optimal background models is also a critical issue, and exerts a strong influence on the relevance of discovered motifs [7]. Background models are generally defined in an organism-specific way, and the divergence of promoter composition across taxa opens the question about the definition of optimal taxon-wide background models.

Despite the wide variety of approaches used to detect phylogenetic footprints, not very much has been done to evaluate their optimal conditions of utilization. In most publications, the quality of the predictions is illustrated on the basis of a few selected transcription factors (between 3 and 10). To our knowledge, a systematic evaluation has been performed in a very few cases. McCue and co-workers [18] evaluated their approach on a complete collection of known motifs in *Escherichia coli*. Evolutionary models, generated by simulating mutations in non-coding sequences, have been used to assess the performances of pairwise [28] or multiple [29] alignment programs. More recently, PhyloGibbs [26] was evaluated on 5 *Saccharomyces *species with 200 annotated TFs, and compared to 6 other algorithms (EMnEM, PhyME, WGibbs and MEME).

Several questions remain open. What is the optimal taxonomical level to detect conserved elements? How to deal with the redundancy between promoters of very close species? How to calibrate taxon-wide background models? Which fraction of existing motifs can we hope to detect (sensitivity)? Which fraction of the discovered motifs can we trust as reliable (predictive value)? How can we select score thresholds to tune up the tradeoff between sensitivity and predictive value?

We address all these questions by evaluating the capability of a pattern discovery algorithm (*dyad-analysis*) to predict cis-acting elements in bacterial promoters. This algorithm is based on the statistical detection of over-represented spaced motifs [9,30], and is particularly well suited for the detection of cis-regulatory motifs in bacterial promoters, since most bacterial transcriptions factors belong to the Helix-Turn-Helix (HTH) family. HTH proteins typically form dimers that bind to spaced motifs [31]. In this context, we use the term *dyad *to denote a pair of trinucleotides separated by a spacing of fixed size but variable content. Another advantage of *dyad-analysis *is that it is based on a significance test, which returns an estimated E-value for each motif, thereby permitting to control the rate of false positives. A third important feature of *dyad-analysis *is that it supports a variety of background models to estimate the prior probability of each dyad.

We first illustrate the approach with a well-characterized case, the auto-regulation of the transcription factor LexA at a single taxonomical level (Gamma-proteobacteria) [32]. We then present a systematic evaluation of our method on 368 *E. coli *genes annotated in RegulonDB [33]. Discovered motifs are evaluated at two levels: correctness and statistical significance. The correctness is estimated by comparing discovered motifs to those previously annotated. The evaluation of significance is based on a comparison between motifs discovered in promoters of orthologous genes (putative footprints), and those discovered in random selections of promoters (spurious motifs). In the last section, we further illustrate the potential interest of our approach by performing a detailed analysis of the auto-regulation of the transcription factor LexA across the whole bacterial taxonomy. Several distinct motifs are detected, each being characteristic of a particular taxonomic class. The correctness of the discovered motifs is confirmed by previously published experiments.

## Results and discussion

### Discovering the LexA auto-regulation in Gamma-proteobacteria

We will illustrate our method by focusing on the gene *lexA*, which codes for the LexA repressor, involved in the SOS response to DNA damage (review in [34]). In *E. coli*, the LexA protein regulates its own production by recognizing specific binding sites in the upstream region of its own gene (*lexA*) [35,36].

**Figure 1:**
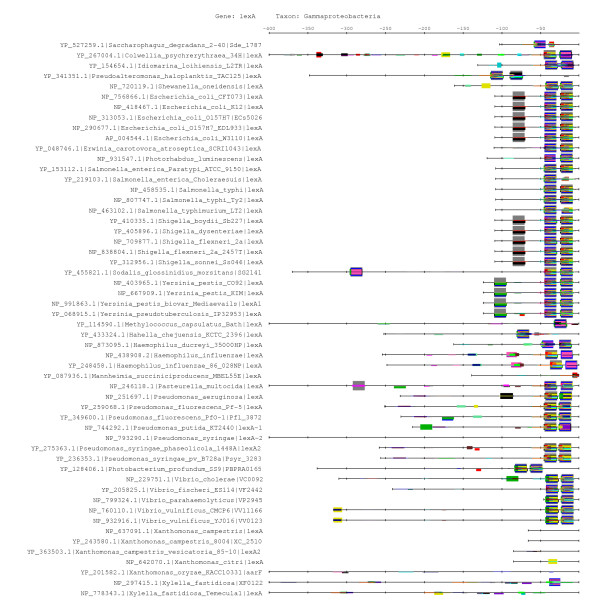
**Feature-map of the dyads detected as significant in *lexA *promoters of Gamma-proteobacteria**. Each line represents one upstream sequence and discovered motifs are displayed on the feature-map as colored boxes with a height proportional to the significance score. The maps displays all dyads with a significance >= 2.

**Figure 2:**
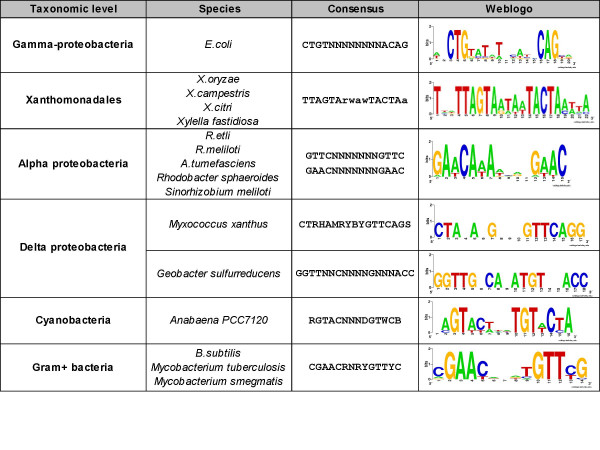
**LexA binding motifs experimentally characterized in different taxonomic groups**. Gamma-proteobacteria [36, 74, 75]; Xanthomonadales [46]; Alpha-proteobacteria [76, 41]; Delta-proteobacteria [77]; Cyanobacteria [78]; Gram-positives [48, 79, 80]. Sequence logos were generated with Weblogo [81] from the alignment of the annotated sites. The *E. coli *sequence logo was obtained from annotated sites in RegulonDB [82, 43].

We identified 181 putative orthologs of LexA in 286 bacterial genomes, and collected upstream non-coding sequences up to the next gene, with a maximal length of 400 bp. The choice of this limit was based on statistical analyses of the TFBS positions in two phylogenetically distant model organisms: *E. coli *[37,38] and *B. subtilis *[39]. Table 1 shows the most significant dyads detected in the 53 promoters of the Gamma-proteobacterial orthologs of *lexA*. Among the 38,615 distinct dyads found in these sequences, 184 are significantly over-represented, among which 122 show a perfect match with the two annotated *lexA *binding sites. On the feature map (Figure 1), these dyads appear aggregated in clumps, thereby revealing different fragments of a same motif. Indeed, the majority of the significant dyads can be assembled to form a larger motif (Table 2), whose strict consensus, A**CTGT**ATATATAT**ACAG**T, corresponds to the LexA consensus (matching nucleotides are in bold) (Figure 2). The most significant dyad, CTGn_10_CAG, corresponds to the most conserved nucleotides of this consensus [36,40,41], and to the interface between the transcription factor and the DNA [42]. This example illustrates the main keys for interpreting the result of dyad-analysis: a motif is generally detected as a collection of mutually overlapping dyads, which can be assembled to form an extended consensus. The most significant dyads of the collection generally correspond to nucleotides entering in direct contact with the transcription factor.

**Table 1:** Most significant dyads detected in promoters of the 53 Gamma-proteobacterial orthologs of *lexA*.

**Dyad sequence**	**Dyad identifier**	**obs_occ**	**expected_freq**	**exp_occ**	**occ_P**	**occ_E**	**occ_sig**	**rank**
ctgn{10}cag	ctgn{10}cag|ctgn{10}cag	45	0.0002971522775	1.88	6.2e-45	2.4e-40	39.62	1
ctgn{9}aca	ctgn{9}aca|tgtn{9}cag	47	0.0005035119746	3.19	2.1e-37	8.2e-33	32.08	2
ctgn{8}tac	ctgn{8}tac|gtan{8}cag	36	0.0003516501339	2.26	2.7e-30	1.1e-25	24.98	3
actn{11}cag	actn{11}cag|ctgn{11}agt	38	0.0004247904187	2.66	4.5e-30	1.7e-25	24.76	4
ctgn{1}ata	ctgn{1}ata|tatn{1}cag	43	0.0005681739096	3.83	6.8e-30	2.6e-25	24.58	5
actn{10}aca	actn{10}aca|tgtn{10}agt	39	0.0004770216061	3.02	2.6e-29	9.8e-25	24.01	6
atan{0}cag	atan{0}cag|ctgn{0}tat	39	0.0004971628517	3.38	1e-27	3.9e-23	22.41	7
tgtn{8}aca	tgtn{8}aca|tgtn{8}aca	31	0.0002748483967	1.76	1.6e-27	6.0e-23	22.22	8
atan{6}cag	atan{6}cag|ctgn{6}tat	40	0.0005662014535	3.68	2.5e-27	9.7e-23	22.01	9
tatn{0}aca	tatn{0}aca|tgtn{0}ata	37	0.0005149051727	3.50	4.2e-25	1.6e-20	19.79	10
ctgn{7}ata	ctgn{7}ata|tatn{7}cag	33	0.0005399616828	3.48	5e-21	1.9e-16	15.71	11
gtan{7}aca	gtan{7}aca|tgtn{7}tac	29	0.0003877441875	2.50	5.6e-21	2.2e-16	15.66	12
atan{1}aca	atan{1}aca|tgtn{1}tat	38	0.0007529946923	5.07	1.2e-20	4.7e-16	15.32	13
atan{2}cag	atan{2}cag|ctgn{2}tat	34	0.0006478755931	4.32	3.2e-19	1.2e-14	13.90	14
ctgn{3}ata	ctgn{3}ata|tatn{3}cag	32	0.0006173317980	4.10	4.2e-18	1.6e-13	12.79	15
atan{0}tac	atan{0}tac|gtan{0}tat	30	0.0005247295752	3.57	5.5e-18	2.1e-13	12.67	16
actn{0}gta	actn{0}gta|tacn{0}agt	26	0.0003805581878	2.59	1.4e-17	5.2e-13	12.28	17
tatn{2}aca	tatn{2}aca|tgtn{2}ata	34	0.0008118555000	5.42	2.4e-16	9.2e-12	11.04	18
tatn{6}aca	tatn{6}aca|tgtn{6}ata	32	0.0007321348527	4.75	2.8e-16	1.1e-11	10.97	19
gtan{2}tac	gtan{2}tac|gtan{2}tac	17	0.0001436260620	0.96	7.1e-16	2.7e-11	10.56	20

The table only displays the 20 dyads having a *sig ≥ 10 *using the TAXFREQ background model. Column content: (1) dyad; (2) dyad identifier (the dyad + its reverse complement); (3) observed number of occurrences (obs_occ); (4) expected frequency (exp_freq); (5) expected number of occurrences (exp_occ); (6) P-values (occ_P), (7) E-value (occ_E), significance (occ_sig), rank.

**Table 2:** Assembly of the patterns discovered in Gamma-proteobacteria.

**Alignment**	**Reverse complement**	**Sig**
act...........cag.	.ctg...........agt	24.76
act..........aca..	..tgt..........agt	24.01
actgta............	............tacagt	12.28
**.ctg..........cag.**	**.ctg..........cag.**	39.62
.ctg.........aca..	..tgt.........cag.	32.08
.ctg........tac...	...gta........cag.	24.98
.ctg...........agt	act...........cag.	24.76
.ctg.ata..........	..........tat.cag.	24.58
.ctgtat...........	...........atacag.	22.41
.ctg......tat.....	.....ata......cag.	22.01
.ctg.......ata....	....tat.......cag.	15.71
.ctg..tat.........	.........ata..cag.	13.90
.ctg...ata........	........tat...cag.	12.79
..tgt.........cag.	.ctg.........aca..	32.08
..tgt..........agt	act..........aca..	24.01
..tgt........aca..	..tgt........aca..	22.22
..tgtata..........	..........tataca..	19.79
..tgt.......tac...	...gta.......aca..	15.66
..tgt.tat.........	.........ata.aca..	15.32
..tgt..ata........	........tat..aca..	11.04
..tgt......ata....	....tat......aca..	10.97
...gta........cag.	.ctg........tac...	24.98
...gta.......aca..	..tgt.......tac...	15.66
...gtatat.........	.........atatac...	12.67
....tat.......cag.	.ctg.......ata....	15.71
....tat......aca..	..tgt......ata....	10.97

a**ctg**tatatatata**cag**t	a**ctg**tatatatata**cag**t	best consensus

The selected dyads (*sig ≥ 10*) can be aligned on the most significant one (CTGn_10_CAG). The "best" consensus of the alignment, obtained by retaining the highest scoring letter at each position, is ACTGTATATATATACAGT.

### Systematic evaluation of the approach

In order to estimate the general performances of the approach, we performed a systematic analysis of the 368 protein-coding genes having annotated TF binding sites in RegulonDB [43], and for which an identifier was found in the Genbank/NCBI genome of the strain *E. coli K12*. This analysis was led for all the ancestral taxonomical levels of *E. coli*: Escherichia (genus), Enterobacteriales (order), Gamma-proteobacteria (class), Proteobacteria (phylum), Bacteria (superkingdom), by increasing order of phylogenetic distance.

#### Impact of the parametric choices: dyad filtering, background model, operon inference, and taxonomical level

Footprint detection involves several crucial parametric choices. A first option, called "dyad filtering", was to consider either the dyads found in all the promoters of the orthologs of the gene of interest, or only those found in the promoter of the reference species (*E. coli K12*). A second option was to use either a gene-specific (MONAD) or a taxon-wide (TAXFREQ) background model. A third option was to collect promoter sequences either directly upstream of each gene, or to infer operons and to retrieve the promoter of their leader gene. A fourth option is the choice of the taxonomical level at which orthologs are collected. In total, there are 40 possible combinations of these 4 parameters. In this section, we present a systematic evaluation of their impact on the accuracy of the predictions.

##### Gene-per-gene comparison

The correctness of discovered motifs was assessed by a gene-per-gene comparison between significant dyads and experimentally characterized binding sites (as annotated in RegulonDB for *E. coli*). A dyad is considered as true positive if it perfectly matches at least one of the annotated sites for the considered gene. For a given pattern discovery result, we define the *sensitivity *(*Sn*) as the fraction of annotated binding sites matched by at least one discovered pattern, and the *positive predictive value *(*PPV*) as the fraction of discovered patterns matching at least one annotated site. The tradeoff between sensitivity and PPV is measured by the computing the geometric accuracy, $Acc_{g}=\sqrt{Sn\cdot PPV}$ For example, in the *lexA *result of the previous section, both annotated sites are matched by at least one dyad, the sensitivity is thus *Sn = 2/2 = 100%*. Among the 184 discovered dyads, 122 match at least one annotated site, the positive predictive power is thus *PPV = 122/184 = 66.3%*. The resulting geometric accuracy is $Acc_{g}=\sqrt{100\%\cdot 66.3\%}=81.4\%$.

**Figure 3:**
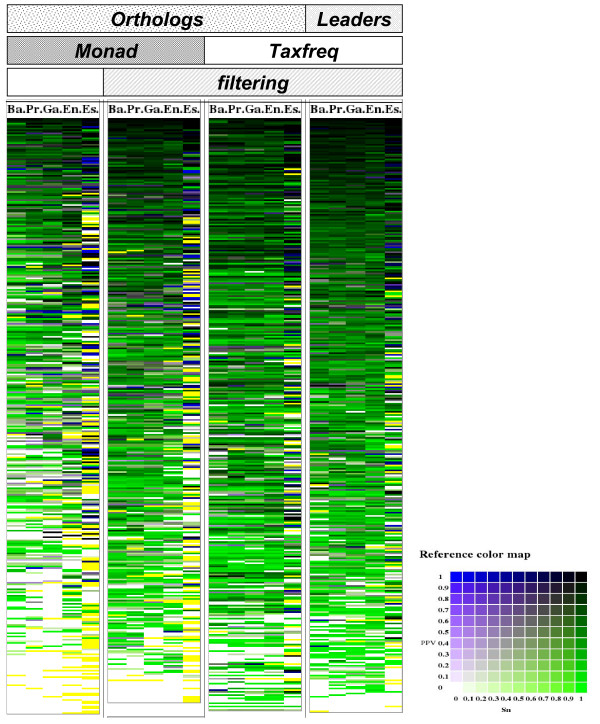
**Correctness of dyads predicted by group of genes and taxonomical level**. Rows represent genes with annotations in RegulonDB (368 genes), and are ordered by sum of geometric accuracy then by maximal geometric accuracy. Different conditions are represented: choice of upstream sequences (orthologs or predicted leader genes), background model (MONAD or TAXFREQ) and the use of dyads filtering or not. For each condition, the first five columns correspond to decreasing taxonomical levels: Bacteria (Ba.), Proteobacteria (Pr.), Gamma-proteobacteria (Ga.), Enterobacteriales (En.) and Escherichia (Es.). The background color reflects the tradeoff between the PPV (blue channel) and the sensitivity (green channel) as shown on the reference colour scale. A perfect prediction is displayed with a black background. When the predictions do not match any annotated sites, the accuracy is zero with a white background. Yellow background corresponds to *NA *which means there is not a single discovered dyad or that the analysis could not be performed (not enough sequences to analyze or too redundant sequences).

Figure 3 summarizes the results obtained with 20 combinations of parameters, each one being depicted as an "accuracy heat map", where rows correspond to groups of orthologs and columns to taxonomical levels (the other combinations are shown in Additional file 1). The darkness is proportional to the accuracy (a perfect prediction is displayed in black), and the color code represents the tradeoff between sensitivity (green) and specificity (blue). Note the overall prevalence of green hues, indicating that the sensitivity is usually higher than the PPV at the default significance threshold (*sig ≥ 0*). Not surprisingly, when applying higher thresholds of significance, the heat maps show a progressive decrease in darkness, reflecting the loss in sensitivity, together with an increased predominance of the blue color, reflecting the increase of predictive value (see Additional file 2). Beyond these general trends, accuracy heat maps show that the optimal taxonomical level can vary from gene to gene. The rightmost column of each parametric condition, corresponding to the genus Escherichia, shows interspersed dark and yellow/white bars, indicating the erratic character of these predictions. The parameter having the strongest impact on the accuracy is the dyad filtering, as denoted by the fact that corresponding heat maps systematically appear darker than those of non-filtered dyads, all other conditions being identical. The color maps also suggest that taxon-wide background models (TAXFREQ) are systematically better than gene-wise models (MONAD).

An important fraction of bacterial genes are organized in operons, i.e. polycistronic transcription units. In such cases, transcriptional regulation is mediated at the level of the promoter of the operon leader gene. Intra-operon intergenic sequences are generally much shorter than real promoters, and this feature has been exploited to predict operons in completely sequenced genomes [40]. We evaluated the impact of operon inference on the quality of the detected footprints: instead of retrieving the sequence directly upstream of each gene, we select the sequence upstream of the leader gene of its predicted operon. On the heat map, operon inference seems to improve the predictions for some genes, and weaken it for other genes, but, based on visual impression, it is hard to evaluate the general effect on the average darkness for all the genes.

##### Quantitative comparison of parameter combinations

In order to quantify the impact of the respective parameters, we averaged the accuracy for all genes in each condition (Table 3), and applied the Wilcoxon paired test (Table 4) to each parameter (dyad filtering, operon inference, background model, and all possible pairs of taxa). The most significant parameter is the choice of the background model (P-value = 9.5E^-7 ^in Table 4). Consistently, Table 3 shows that taxon-wide background models (TAXFREQ) give systematically better results than gene-wise models (MONAD), all other parameters being identical. The second parameter, dyad filtering, also shows a straightforward effect (P-value = 4.8E^-5^): the accuracy is systematically improved when dyad filtering is applied. By contrast, operon inference gives variable results, depending on the other parameter values: retrieving the promoter from the operon leader gene gives better results in 5 cases, but worse results in 13 other cases (Table 3). Indeed, the high P-value (10.7%) indicates that this parameter is poorly significant. The poor impact of operon prediction might be influenced by the fact that we analysed genes with known sites in their promoter region in *E. coli K12 *(these genes are thus always operon leaders, at least in the reference organism). However, operon inference might improve the results of the analysis of all genes of a genome for which there would be no prior knowledge on the motifs.

**Table 3:** Impact of the parametric choices on the correctness of the discovered motifs

**Rank**	**promoter**	**bg**	**filtering**	**Taxon**	**Acc_g_**	**Sn**	**PPV**
1	ortho	taxfreq	filtered	Gammaproteobacteria	0.658	0.844	0.513
2	leader	taxfreq	filtered	Gammaproteobacteria	0.657	0.844	0.512
3	ortho	taxfreq	filtered	Proteobacteria	0.647	0.840	0.499
4	leader	taxfreq	filtered	Proteobacteria	0.642	0.844	0.488
5	ortho	taxfreq	filtered	Enterobacteriales	0.637	0.857	0.473
6	ortho	taxfreq	filtered	Bacteria	0.634	0.799	0.503
7	leader	taxfreq	filtered	Enterobacteriales	0.631	0.860	0.463
8	leader	monad	filtered	Proteobacteria	0.622	0.798	0.484
9	leader	taxfreq	filtered	Bacteria	0.621	0.796	0.485
10	leader	monad	filtered	Gammaproteobacteria	0.607	0.757	0.487
11	ortho	monad	filtered	Proteobacteria	0.605	0.76	0.482
12	ortho	monad	filtered	Gammaproteobacteria	0.598	0.742	0.481
13	ortho	monad	filtered	Bacteria	0.596	0.778	0.457
14	leader	monad	filtered	Bacteria	0.593	0.810	0.433
15	leader	monad	filtered	Enterobacteriales	0.548	0.714	0.420
16	ortho	monad	filtered	Enterobacteriales	0.544	0.706	0.419
17	ortho	taxfreq	filtered	Escherichia	0.533	0.626	0.453
18	leader	taxfreq	filtered	Escherichia	0.528	0.626	0.445
19	ortho	taxfreq	no	Escherichia	0.525	0.582	0.473
20	leader	taxfreq	no	Escherichia	0.518	0.591	0.453
21	leader	taxfreq	no	Gammaproteobacteria	0.500	0.694	0.360
22	leader	taxfreq	no	Enterobacteriales	0.497	0.736	0.335
23	ortho	taxfreq	no	Enterobacteriales	0.495	0.735	0.333
24	ortho	taxfreq	no	Gammaproteobacteria	0.488	0.706	0.338
25	leader	monad	no	Enterobacteriales	0.480	0.574	0.402
26	ortho	monad	no	Enterobacteriales	0.480	0.573	0.402
27	ortho	taxfreq	no	Proteobacteria	0.463	0.738	0.291
28	leader	taxfreq	no	Proteobacteria	0.461	0.719	0.295
29	ortho	monad	no	Gammaproteobacteria	0.453	0.545	0.376
30	leader	monad	no	Gammaproteobacteria	0.451	0.551	0.370
31	ortho	taxfreq	no	Bacteria	0.435	0.644	0.294
32	leader	monad	no	Proteobacteria	0.428	0.605	0.303
33	ortho	monad	no	Proteobacteria	0.428	0.564	0.324
34	leader	taxfreq	no	Bacteria	0.418	0.634	0.276
35	ortho	monad	filtered	Escherichia	0.397	0.391	0.403
36	leader	monad	filtered	Escherichia	0.396	0.402	0.390
37	ortho	monad	no	Escherichia	0.384	0.366	0.403
38	leader	monad	no	Escherichia	0.383	0.373	0.395
39	ortho	monad	no	Bacteria	0.367	0.607	0.222
40	leader	monad	no	Bacteria	0.341	0.631	0.184

**Table 4:** Significance of the impact of individual parameters on the accuracy of discovered motifs

**Parameter**	**Better**	**Worse**	**N**	**Sup**	**Inf**	**Equal**	**P-value**
**Background model**	taxfreq	monad	20	20	0	0	9.54E-07
**Dyad filtering**	filtered	not filtered	20	20	0	0	4.78E-05
**Taxon**	Gammaproteobacteria	Bacteria	8	8	0	0	0.004
**Taxon**	Proteobacteria	Bacteria	8	8	0	0	0.004
**Taxon**	Enterobacteriales	Escherichia	8	6	2	0	0.020
**Taxon**	Gammaproteobacteria	Escherichia	8	6	2	0	0.020
**Taxon**	Gammaproteobacteria	Proteobacteria	8	6	2	0	0.034
**Taxon**	Proteobacteria	Escherichia	8	6	2	0	0.070
**Taxon**	Enterobacteriales	Bacteria	8	6	2	0	0.074
**Promoter**	orthologs	operon leader	20	13	5	2	0.107
**Taxon**	Bacteria	Escherichia	8	4	4	0	0.191
**Taxon**	Gammaproteobacteria	Enterobacteriales	8	5	3	0	0.273
**Taxon**	Enterobacteriales	Proteobacteria	8	4	4	0	0.528

Parameters are sorted according to the significance of their impact on the geometric accuracy. The best parameter value of each pair is in the left column. N: number of parametric combinations. Sup, inf, equal: number of pairs where the left value gives better, wose or identical results than the right value, respectively. The P-value is estimated with a Wilcoxon paired test.

For the choice of the optimal taxonomic level, we performed pairwise comparisons between each possible pair of taxa considered in this study. The class Gamma-proteobacteria appears as the optimal taxonomical level, since it systematically appears in the left column, indicating that it gives better results than any other taxon. On the contrary, analyses restricted to the various strains of the same genus (*Escherichia*) give systematically worse results that the other taxa.

In summary, Tables 3 and 4 clearly show that the best results are obtained when phylogenetic footprints are discovered at the level of Gamma-proteobacteria, with taxon-wide background model, and with species-specific dyad filtering, and that operon inference has no obvious impact on the general accuracy.

#### Correctness of the discovered patterns

**Figure 4:**
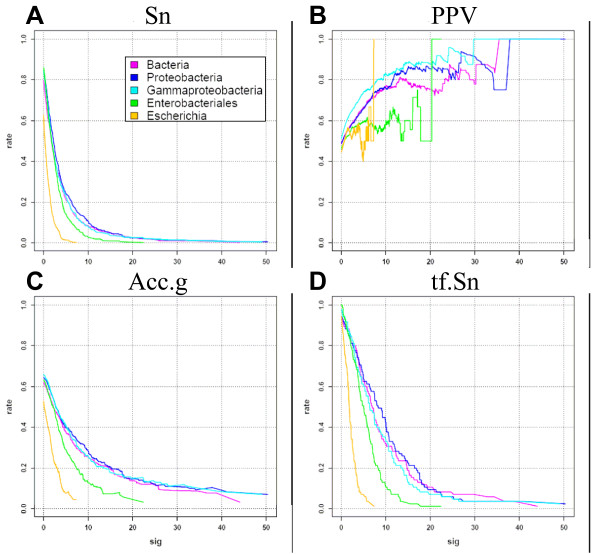
**Systematic evaluation of the performances obtained with the TAXFREQ background model using the upstream sequences of the predicted leader genes and the dyads filtering**. Gene-wise sensitivity (A), PPV (B), geometric accuracy (C), and transcription factor-wise sensitivity (D).

The correctness of predictions was estimated by comparing discovered motifs with annotated binding sites. Figure 4 displays the inverse cumulative distribution of sensitivity, PPV and Accuracy as a function of the significance threshold. As expected, the PPV increases with the significance score (Figure 4B), at the expense of sensitivity (Figure 4A). There is also a clear impact of the taxonomical level on the matching statistics: the sensitivity is higher at the level of the class (Gamma-proteobacteria) or above (Proteobacteria, and Bacteria) than at the level of the order (Enterobacteriales) or below (Escherichia). At the closest level (genus Escherichia), the PPV curve is very erratic, because a very few motifs are detected, due to the small number of species included in the analysis and the low level of divergence between those genomes. The tradeoff between sensitivity and PPV is measured by the geometric accuracy (Figure 4C). The optimal geometric accuracy is obtained around the default significance threshold (*sig ≥ 0*) for all taxonomical levels (not shown). The accuracy curves are lower for narrow taxonomical levels (Enterobacteriales and Escherichia) than for wider groups (Gamma-proteobacteria to Bacteria). The maximal geometric accuracy is reached for Gamma-proteobacteria (64.8%) with a sensitivity of 86.4% and a PPV of 48.7% at the default threshold. The choice of the threshold should however not be based on the sole criterion of optimal accuracy, but can depend on the purpose of the analysis. For example, selecting the motifs with a very high significance (e.g. *sig ≥ 10*) will result in a drastic loss of sensitivity (10%), but is likely to return the most promising motifs (PPV = 80%) as candidates for experimental validation.

Figure 4D displays the transcription factor-wise sensitivity, defined as the fraction of transcription factors for which the correct motif has been found in at least one target gene. This figure shows the same trends as the gene-wise sensitivity (Figure 4A), and indicates that, with the default significance threshold, the correct motif can generally be detected in at least one of the target genes for each transcription factor, and this at any taxonomical level.

For 14 genes among 368, our approach failed to return any true positive. We identified two reasons for these failures. The first reason is the absence of the motif from the promoter sequences analyzed. This absence is in some cases due to errors in the annotated position of the start codon (as discussed below for some Xanthomonadales). In some other cases, a cis-acting element is actually located downstream of the start codon, in the beginning of the coding sequence of the regulated gene. Intragenic regulatory are generally involved in transcriptional repression, and a few such cases are reported in RegulonDB, and thus included in our testing set (see Supplementary material). It has previously been reported that 71% of the promoters contain at least one repressing site downstream of the transcription start site, whereas there is not a single annotated example of downstream activating site [38]. In some cases, binding sites are even found in the coding sequence, downstream of the start codon. As example, Yang et al [44] characterized three binding sites for the repressor GalR in the coding region of the gene *glpT *which codes for a transporter of the glycerol-3-P.

Another reason for not detecting the correct motifs comes from genes having only a small number of orthologs, when the analysis is restricted to a very narrow taxon (e.g. the genus Escherichia). In such cases, the promoters are largely redundant, and functional sites are masked during the purging procedure.

#### Reliability of the significance score

In the previous chapter, we evaluated the accuracy of pattern discovery by comparing discovered motifs with those annotated in the reference database, RegulonDB. However, the concept of false positive is not trivial, since the reference set cannot be considered as exhaustive, for two reasons: (1) the annotation effort requires time, so that the regulatory elements stored in the database onlyrepresent a subset of the published ones; (2) despite de fact that *E. coli *has been, since several decades, among the most popular model organisms for hundreds geneticists and molecular biologists, it is clear that a good fraction of its regulation has still not been characterized.

In this section, we present a simple strategy for assessing the reliability of the significance without depending on any prior annotation about cis-regulatory elements. This strategy relies on a comparison of scores distributions obtained in a positive and a negative sequence set, respectively.

As discussed elsewhere [7,9] the significance score returned by *oligo-analysis *and *dyad-analysis *is the minus-log of the binomial E-value, and can directly be interpreted in terms of risks of false positive: for a given value *sig = s*, the expected number of false positives is *10*^-*s*^. For example, a threshold *sig ≥ -2 *corresponds to an E-value of 100, meaning that, in a random data set, one would expect an average of 100 dyads to be selected by chance with such a score (and thus 100 false positives). Similarly, one would expect an average of 1 dyad per random set with a threshold *sig ≥ 0*, and 0.01 dyad per random set with a threshold *sig ≥ 2*. The E-value provides thus a very intuitive indication of the reliability of the discovered motifs (the lower the E-value, the more reliable are the motifs).

A first question is whether this theoretical significance score corresponds to the rate of false positives observed in a random data set. Indeed, the theoretical model underlying the estimation of E-value is based on some assumptions about sequences (in short, the working hypothesis is that sequences can be modelled as homogeneous Markov chains). A second question is whether the significance score makes an efficient distinction between biologically relevant and spurious motifs.

**Figure 5:**
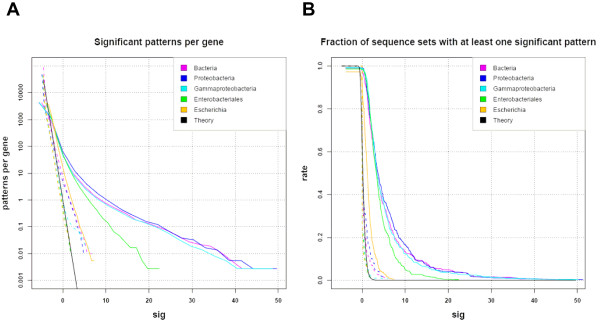
**Distribution of significance score values returned by *dyad-analysis *in promoters of orthologous clusters (solid lines) or in random selections of genes (dotted lines)**. Each curve represents the results of the analysis at one taxonomical level. A. Number of dyads per sequence set. B. Number of sequence sets with at least one significant motif. Black lines represent the theoretic curves. We run *dyad-analysis *with the TAXFREQ background model and the *E. coli*-specific dyads filtering.

Figure 5 shows the inverse cumulative distribution of significance scores obtained in promoters of orthologous genes (positive sets, plain lines) and in random selection of promoters (negative control, dotted lines), respectively.

The left panel (Figure 5A) shows the expected and observed number of significant patterns per gene (ordinate) above each significance value (abscissa). The black curve represents the expected number of false positives per data set, on the basis of the theoretical model. This corresponds to the E-value, which, by definition, decreases exponentially with increasing significance values (E-value = 10^-sig^), and thus appears as a straight line on the graph with a logarithmic ordinate.

The negative control consists in running the footprint detection in sequences where there are supposedly no conserved motifs. For this, we perform random selections in the complete collection of bacterial promoters at the considered taxonomical level. If the background model is correct, the empirical rate of false positives should fit with the theoretical curve. This is clearly the case for the three lowest taxonomical levels (Escherichia, Enterobacteriales and Gamma-proteobacteria), whereas the rate of false positives is higher for larger taxa (Proteobacteria and Bacteria). This increase in the rate of false positives indicates that the background model is not perfectly appropriate for large taxonomical level, probably due to the fact that these promoters contain mixtures of G+C-rich and G+C-poor genomes (the mixture of sequences thus correspond to an heterogeneous Markov model).

The positive test consists in measuring dyad significance in sequences supposed to contain conserved motifs, i.e. promoters of orthologous genes (plain lines on Figure 5). There is a clear effect of the taxonomical level, and the general trend is that the number of significant patterns increases with broader taxonomical levels.

The difference between the negative and positive curves indicates the reliability of the significance score to distinguish relevant (orthologous) from irrelevant (random) gene sets. At the narrowest taxonomical level (Escherichia), the two curves are very close from each other, and from the theoretical curve. This weak significance reflects the insufficient signal-to-noise ratio, due to the small number of available sequences (5 genomes), and to the fact that they are too similar with each other (5 strains of the same species). The number of detected patterns already increases at the level of the order (Enterobacteriales), but the highest sensitivity is clearly obtained at the level of the class (Gamma-proteobacteria) and above (Proteobacteria and Bacteria). The best signal-to-noise ratio is obtained at the level of the class (Gamma-proteobacteria), where the random curve is very close to the theoretical expectation, whereas the number of patterns found in orthologous groups is almost as high as at the highest taxonomical levels.

Figure 5B displays the fraction of genes for which at least one pattern has been returned. This curve is obtained by selecting, for each data set, the top scoring dyad only. The theoretical curve (black curve) is estimated from a Poisson distribution, and reflects the Family-Wise Error Rate (FWER), i.e. the probability to have at least one false positive dyad in a random set. The negative (dotted lines) and positive (plain lines) show the same trends as in Figure 5A.

In summary, our analysis shows that the E-value returned by dyad-analysis gives a reliable estimate of the expected number of false positives, and that it clearly distinguishes phylogenetic footprints from spurious motifs.

### Deciphering the evolution of LexA auto-regulation

The analyses above show that cis-acting elements can be detected by their conservation across the promoters of a set of related species. A complementary question is whether a separate analysis of distant taxonomic groups would allow us to detect divergences between regulatory motifs. To address this question, we focused our attention on the LexA regulator, whose peptidic sequence is highly conserved across bacteria, and for which distinct DNA-binding motifs have been characterized for various bacterial taxa (Figure 2), suggesting a co-evolution of the transcription factor with its DNA binding motif [45]. The question addressed in this section is whether footprint discovery would allow us to rediscover the evolution of LexA auto-regulation.

**Figure 6:**
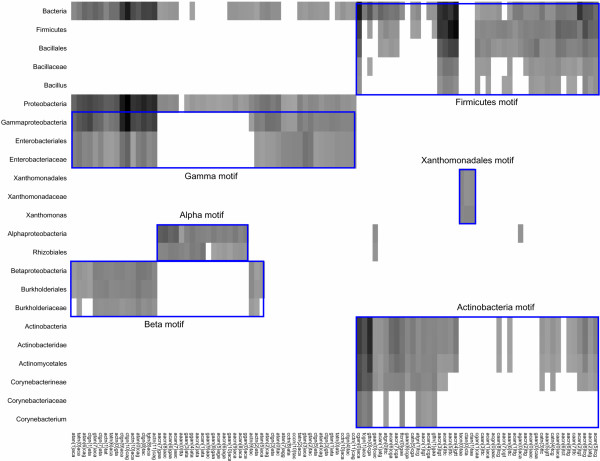
**Significance heat map of the dyads discovered at different taxonomical levels in upstream sequences of lexA orthologs**. Each column represents one pattern, each row one taxon. The grey level indicates the significance score returned by *dyad-analysis*: black corresponds to the maximal score (*sig = 41.01*) obtained in the whole taxonomy, and non-significant patterns appear with a white color. Patterns are clustered by sequence similarity (Ward linkage hierarchical clustering). Taxa are ordered by depth-first-search traversal of the taxonomic tree. In order to simplify this heat map, we used a stringent threshold, by only selecting taxa having at least one predicted pattern with a significance score ≥7. We manually boxed groups of patterns corresponding to taxon-specific motifs.

Figure 6 shows a synthetic view of the most significant dyads discovered at each taxonomical level. This figure is displayed in Additional file 3 with the tree corresponding to the dyads clustering. For the sake of clarity, we selected dyads with a high significance level (sig >= 7). This view illustrates the taxon specificity of the detected motifs: distinct clusters of dyads are detected at the different taxonomical levels, with a strong consistency: a group of 35 dyads is found, with various levels of significance, at all taxonomical levels from Enterobacteriaceae to Bacteria (Gamma-proteobacterial motif). A subset of these is also found in Beta-proteobacteria. However, completely distinct motifs are found in Alpha-proteobacteria and Xanthomonadales, respectively. Interestingly, the hierarchical clustering of motifs by sequence similarity clearly corresponds to taxonomical divergences: the motifs found in the left part of Figure 6 are those discovered in Proteobacteria (Alpha-, Beta- and Gamma-proteobacteria) whereas the right side regroups motifs detected in Gram-positive bacteria (Firmicutes and Actinobacteria). The only exceptions are the motifs found in Xanthomonadales, which are grouped with Gram-positive bacteria, whereas this order belongs to Gamma-proteobacteria (detailed analysis below).

**Figure 7:**
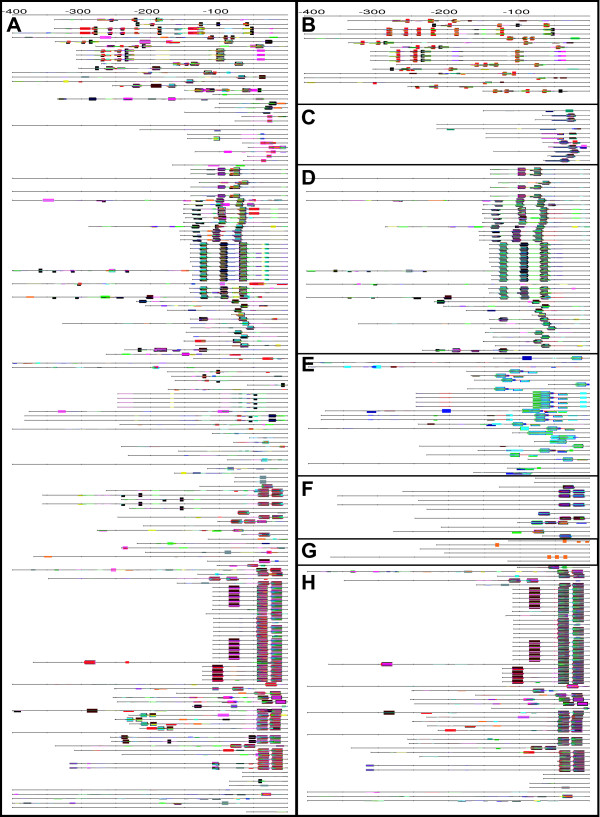
**Feature-maps of the dyads detected in promoters of *lexA *orthologs at different taxonomical levels**. In the left hand column, motifs were discovered on the whole collection of bacterial promoters of *lexA *orthologs (A). The right hand column shows motifs detected in subsets corresponding to narrower taxa: Actinobacteria (B), Cyanobacteria (C), Firmicutes (D), Alpha-Proteobacteria (E), Beta-Proteobacteria (F), Delta-Proteobacteria (G), Gamma-Proteobacteria (H). The maps displays all dyads with a significance >= 2.

Figure 7 shows the localization of the motifs discovered at the level of the Reign (all Bacteria together, left panel) and in each separated class (right panel). This comparison shows that the motifs discovered in the most represented classes (Gamma/Beta-proteobacteria, Firmicutes, Actinobacteria) are also discovered when all the bacterial sequences are analyzed as a single group. This illustrates the robustness of the method to noise, since it can detect motifs presents in a subset of the sequences only. However, some other motifs are significant at the level of the class (e.g. Cyanobacteria, Alpha-proteobacteria) but are not detected when all bacterial promoters are analyzed as a single set. In order to analyze the divergence between regulatory elements, it is thus worth applying pattern discovery at all levels of the taxonomical tree.

#### Comparison of significant dyads with the annotated LexA binding motifs for different taxa

**Figure 8:**
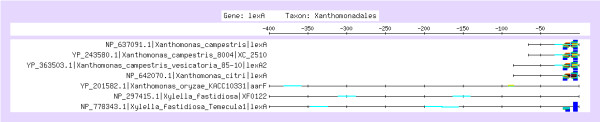
**Feature-map of the dyads detected as significant in promoters of Xanthomonadales**. The symmetrical motif corresponds to the Xanthomonadales-specific LexA binding site regulatory element (see text). Note that, although the motif seems to be missing in two promoters, in both cases it can be found downstream of the annotated start codon (see Supplementary Material), and their absence on the feature map probably reflects errors in the annotation of the start codon, or the presence of a repression site inside the coding sequence (not shown).

As discussed above, the most significant dyads detected in Gamma-proteobacterial promoters (Figure 1) correspond to the LexA binding element characterized in several species of this class (Figure 2). An informative characteristic about discovered motifs is their gene coverage, i.e. fraction of genes whose upstream sequence contains at least one occurrence of the motif. Figure 1 shows that the Gamma-proteobacterial motif is found in 47 among the 54 promoters. Interestingly, the 7 promoters where the motif is absent belong to the order Xanthomonadales (which includes the two genus *Xanthomonas *and *Xylella*). This suggests that LexA auto-regulation might have diverged in this subgroup. Consistently, when pattern discovery is restricted to Xanthomonadales sequences, another motif, TTAGTA is detected in 5 of the 7 promoters. This hexamer is found in two occurrences per sequence, forming an inverted repeat (Figure 8). The assembly of the Xanthomonadales-specific dyads (Table 5) corresponds to the experimentally validated motif [46].

**Table 5:** Evolution of LexA binding motif: comparison between annotated and predicted motifs.

**Taxon**	**Annotated motif**	**Predicted motif**	**Most significant dyads**		**Genes**			**Dyads**	
			
			**pattern**	**sig**	**nb**	**match**	**coverage**	**tested**	**significant**
*Alpha proteobacteria*	**GAAC**nnnnnnn**GAAC**	ttg**gAAC**acataga**gAAC**aga	AACn_8_AAC	18.64	26	24	92%	33800	125
*Beta proteobacteria*	-	ca**cTG**TATAattATA**CAG**tg	ATACAG	11.12	14	9	64%	21649	14
*Gammaproteobacteria*	**CTGT**nnnnnnnn**ACAG**	a**CTGt**atatatat**aCAG**tt	CTGn_10_CAG	38.67	54	44	81%	39229	142
*Xanthomonadales*	**TTAGTArwa**w**TACTAa**	g**TTAGTAata**c**TACTA**Ac	TACTAA	7.5	7	5	71%	15535	76
*Delta proteobacteria*	GGTTnnCnnnnGnnnACC CT**RHAMRY**BYGTTCAGS	**GTAAGT**	GTAAGT	3.98	6	3	50%	17622	3
*Cyanobacteria*	R**GTAC**nnnD**GTWC**B	ag**gtAC**Ata**TGTacc**t	ACAn_2_TGT	6.06	11	7	64%	13696	50
*Firmicutes*	**CGAACR**n**RYGTTY**C	ta**cgAACa**t**atGTTc**gta	AACn_4_GTT	38.79	40	39	98%	31695	140
*Actinobacteria*	**CGAACR**n**RYGTTY**C	aaT**CGAACa**c**atgtTC**GAACat	TCGAAC	30.95	17	17	100%	34155	139

For each analysis at a given taxonomical level (column 1), the annotated motif (column 2) is compared with the predicted motif (column 3) with matching letters in bold. Capital letters in the predicted motif correspond to the most significant dyad (column 4, with significance score in column 5). The gene coverage is the fraction of genes whose upstream sequence contains at least one occurrence of the most significant dyad. The last columns indicate the number of tested dyads and the number of dyads detected as significant (sig > 0), respectively.

When the analysis is restricted to a very narrow taxonomical level, the pattern discovery approach fails to detect motifs, because the species are too close from each other to have allowed divergence between promoter sequences (Mycobacterium tuberculosis complex, Brucellaceae, pseudo mallei group and Bacillus cereus group). In all those cases, the sensitivity of the method is expected to increase with the number of orthologs, so that the missing motifs will hopefully be detected in the future, when additional genomes will be available for these taxonomic groups.

In addition to the 7 taxa where discovered motifs match the annotated one, a highly significant motif is detected in Beta-proteobacteria (Table 5). This motif is the same as for Gamma-proteobacteria, and corresponds to a LexA binding site suggested for Beta-proteobacteria after *in silico *analysis in *Ralstonia solanacearum *[47]. It is detected in 9 of the 14 promoters analyzed (Figure 7F), but, as discussed above for Xanthomonadales, the five remaining genes have one or two occurrences in the beginning of their coding sequences.

In summary, the phylogenetic footprint detection reveals all the taxon-specific LexA binding motifs that had previously been characterized experimentally (Table 5).

#### Divergence of the LexA binding motif in Gram-positive bacteria

**Figure 9:**
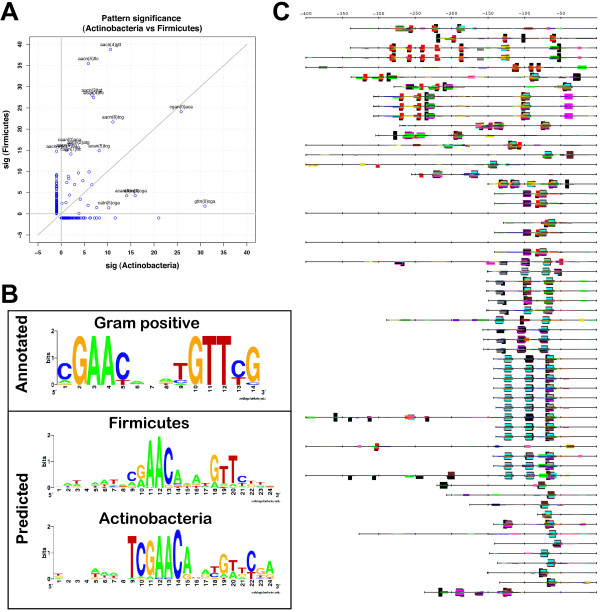
**Partial divergence between the motifs discovered in lexA promoters of Gram-positive bacteria (Firmicutes and Actinobacteria)**. (A) Comparison between the significance scores of all discovered patterns in Actinobacteria and Firmicutes. The significance is arbitrarily set to -1 for non significant patterns (sig < 0), in order to highlight dyads detected in only one of the subgroups. (B) Sequence logo of motifs obtained by aligning all the sites annotated for Gram-positive bacteria (top), Firmicutes (middle) and Actinobacteria (bottom), respectively. For each taxon, all sites matching the discovered patterns were extracted with their flanking region and were aligned using MEME [3] with a motif width of 24 nucleotides. Sequence logos were generated with Weblogo [81]. (C) Feature-map of the dyads discovered in Gram-positive bacteria. The 17 first top sequences are promoters of sequences from Actinobacteria, while others are from Firmicutes. In Actinobacteria, the 11 first sequences are promoters of orthologous sequences from Corynebacterineae.

The relationships between the motifs discovered at different levels of the taxonomic tree can give us insight into evolutionary events, such as conservation, appearance, loss and divergence of regulatory signals. This is well illustrated by the comparison of motifs discovered in Gram-positive bacteria (Figure 9), which are comprised of two phyla: Firmicutes and Actinobacteria. The dyads selected in these two phyla show a partial overlap (Figure 9A,C), but also some interesting differences. The most significant dyads discovered in Firmicutes are AACn_4_GTT and AACn_5_TTC which correspond to the core of the Cheo box G**AAC**n_4_**GTTC **[48] and to the LexA consensus (CG**AAC**RNRY**GTT**YC) proposed for Gram-positive bacteria altogether [49]. In Firmicutes promoters, the most significant dyads are spaced motifs, which can be combined to form a palindromic consensus TAC**GAAC**ATAT**GTTC**GTA. This motif is found in 1 to 3 occurrences per promoter, at more or less conserved positions (Figure 7D). In contrast, the most significant dyads discovered in Actinobacteria are hexanucleotides that can be aligned to form the heptanucleotide TCGAACA (Figure 9A). A comparison of the sequence logos (Figure 9B) shows that this heptanucleotide matches one half of the spaced palindrome found in all the Gram-positive bacteria, but that the half-motif is more specific for Actinobacteria than Firmicutes.

Consistently, the feature map obtained by merging all the gram-positive bacteria (Figure 9C) shows that in Firmicutes, instances of the heptanucleotide generally overlap with the larger Cheo boxes. The situation however differs in Actinobacteria: although some promoters contain one occurrence of the Cheo box, many additional instances of the short motifs are found more distally, without being part of a large motif (Figure 7A). This feature is particularly marked in Corynebacterium (11 first sequences in Figure 9C), where the large motif has apparently been lost, and is somehow "compensated" by a more occurrences of the short motif. This observation raises the intriguing hypothesis that LexA binding specificity is on its way to diverge between Firmicutes and Actinobacteria. The reverse complementary symmetry of a binding motif generally reflects the binding of the transcription factor as a homodimer. This is the case for the Gamma-proteobacterial LexA protein. The symmetry of the large motif has previously been shown in *B. subtilis *[48] and suggests that, in Firmicutes as well, LexA may act as homodimer, and this hypothesis is compatible with the structural model [50]. In Actinobacteria, the imperfect palindromic nature of the SOS box has been previously observed in *recA *promoter of *Streptomyces lividans *[51]. The presence of half-instances of the motif in Actinobacteria opens the question of the structure of the protein/DNA interface. Is it possible for the Actinobacterial LexA to bind DNA as a monomer? Does it form heterodimers with another protein? A structural characterization of the LexA/DNA complex would be required elucidate this mechanism.

Beyond the particular case of LexA auto-regulation, this example shows that the comparative analysis of taxon-specific footprints is a powerful method to trace the evolution of DNA recognition by transcription factors.

### Comparison between dyad-analysis and other pattern discovery algorithms

Several previous studies have addressed similar issues as we do in this paper. In particular, the detection of phylogenetic footprints in bacterial promoters has been treated by several other groups, using various implementations of a *gibbs *sampler [17,52,19].

Pattern discovery algorithms can be broadly subdivided into two main categories: (1) enumerative approaches based on the detection of over-represented words (oligonucleotides or spaced pairs) [7,9,53]; (2) matrix-based approaches, based on various optimization strategies: expectation-maximization [3], greedy algorithm [1], gibbs sampling [2,4,54,11].

#### Reliability of matrix scores

The comparison of score distributions between orthologs and random gene selections presented above can easily be applied to other programs, and used as a criterion to estimate the reliability of the motifs discovered under various conditions.

For obvious reasons, a comparative assessment should in principle not be led by the team who developed one of the programs: a fair comparison should either be done by a naïve user who did not contribute to any of the tools assessed [55], or by letting developers run their own tool on some benchmark data set [56]. However, as a condition for publishing this paper, the referees and editors asked us to compare our own program with other pattern discovery programs. We performed this comparison as honestly as possible, and we tried to select, for each program, the most appropriate parameters. However, it is clear that, even with the best will, there is one bias that cannot be avoided: each developer better knows how to use his/her own tools. It is thus likely that the other programs would return better results in the hands of their respective developers than in ours.

One further remark: as shown in Table 3, dyad filtering has a massive effect on the accuracy of footprint detection. However, the other matrix-based pattern discovery tools do not support the concept of filtering (i.e. the fact to select motifs found in the promoter of a given reference organism, *Escherichia coli *in our case). Thus, for the sake of fairness, we decided to avoid dyad filtering for this comparison, so that all programs are fed with exactly the same sequences, without any prior information on the reference organism. It should be stressed that these conditions are far from our optimum, since they correspond to an accuracy of 48.8%, whereas our best parametric conditions (with filtering) reach an accuracy of 65.8% (Table 3). As shown below, even under these sub-optimal conditions, dyad-analysis outperforms the other programs.

**Figure 10:**
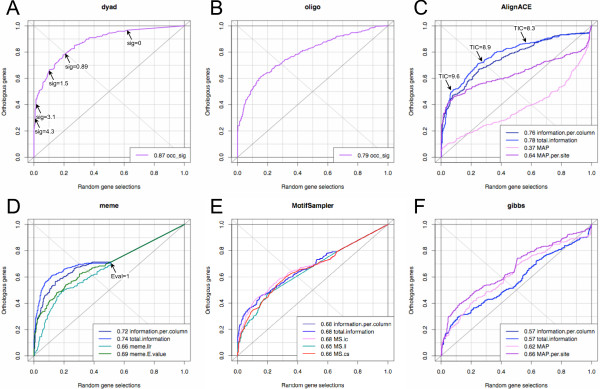
**Comparison between programs and between scoring statistics**. The "ROC-like" curves are displayed for each program: (A) dyad-analysis, (B) oligo-analysis, (C) AlignACE, (D) MEME, (E) MotifSampler and (F) Gibbs sampler.

The results of our comparative assessment are summarized on Figure 10, in the form of "ROC-like" curves. Each curve shows the performances of a given program and a given scoring scheme. For each score value, the curve indicates the fraction of sequence sets with at least one significant motif in promoters of random groups of genes (abscissa) and orthologous genes (ordinate), respectively. For example, Figure 10A indicates that, with a significance threshold of 1.5, motifs are found in 10% of the random selections versus 65% of the orthologous groups. There is thus a "cost" of 10% of false positives for obtaining 65% of sensitivity. With score of 3.1, the rate of false positives drops to 1.4%, but the sensitivity is reduced to 40%.

A classical statistics for estimating the general performances is to compute the Area Under the Curve (AUC). A perfect program would give an AUC of 1.0 (the curve would actually follow the left axis from bottom to top, and then the top axis from left to right), whereas a random predictor would follow the diagonal from bottom left to top right, since it would return as many patterns in random selections as in orthologous groups (the AUC would be 0.5). The sig score of *dyad-analysis *(Figure 10A) gives an AUC of 0.87. By comparison, the word-based pattern discovery program *oligo-analysis *[7] gives an AUC of 0.79 (Figure 10B). Since these two programs use exactly the same binomial statistics for scoring motif significance, the difference comes from the motif structure (dyads versus words) thereby confirming the importance of using spaced motifs to decipher bacterial regulation, as discussed in the introduction.

We also tested four popular matrix-based pattern discovery programs (Figure 10C–F; the supplementary material provides the parameters used for each program). Each of these programs returns several scores per matrix, shown as separate curves on the same plot. In addition to the matrix scores returned by the programs, we computed the total information content (TIC) of each matrix, following to Hertz and Stormo's definition [8], as well as an information per column, in order to discard the effects of matrix width. Interestingly, the total information content (TIC) performs generally better than the original statistics of those programs. This is particularly obvious for AlignACE (Figure 10C), for which the TIC gives an AUC of 0.78, whereas the MAP per site only gives 0.64, and the MAP per matrix gives an AUC smaller than 0.5. Surprisingly, the original gibbs motif sampler [4] shows the opposite trends (Figure 10F): the MAP (AUC = 0.62) gives better results than the TIC (AUC = 0.57). An interesting property of MEME (Figure 10D) [57] is that it returns a negative answer (no matrix) for 50% of the random selections, thanks to the threshold on E-value < 1. However, with this threshold, a negative answer is also returned for 30% of the groups of orthologs. The TIC obtained with MEME matrices gives a total AUC of 0.72, but, if one only considers the subset of sequences giving at least one matrix, the curve shows a pretty good shape.

It should be noted that the AUC, despite its popularity as measure of global performances, can be very misleading since it is integrated over the whole curve, whereas in realistic conditions, a user would not even consider the motifs returned in the right part of the curve, which are expected to return more than 50% of false positives. Beyond the AUC, a more relevant criterion for estimating the relevance of a program is the sensitivity obtained for a given rate of false positives (e.g. 10%). Figure 10 shows that, with a 10% rate of false discovery (random gene selections) *dyad-analysis *discovers motifs in 65% or the orthologous groups, which outperforms MEME TIC (58%), AlignACE TIC (52%), *oligo-analysis *significance (51%), MotifSampler information content (41%), and *gibbs *MAP per site (36%). The program ranking is similar with a rate of false positive of 5%.

In summary, in our hands at least, *dyad-analysis *outperforms the other pattern discovery programs in its capability to discovery higher scoring motifs in promoters of orthologous genes than in randomly selected promoters. We would however like to make clear that we consider this analysis as biased by our better experience for using our own program. Since all the data used for our analyses are available on the supplementary web site, anyone interested is able to perform his/her own tests in order to estimate the most appropriate program.

### Comparison with previous studies

We further discuss hereafter our methodological choices and compare them with previous studies.

#### Prior versus posterior probability of motif occurrences

Many previous studies about footprint discovery in Bacterial promoters were based on various implementations of the Gibbs sampling [17,52,19,26]. This algorithm is based on the optimization of "relative entropy" score [2]. This score is however not efficient to discriminate between informative and non-informative motifs. For this reason, motifs are generally filtered *a posteriori *on the basis of the MAP (*Maximum A Posteriori Probability*) score [54,58].

An advantage of the enumerative approach used here is that the probability of each motif is estimated *a priori *rather than *a posteriori*, on the basis of its occurrences in a reference data set (the complete set of promoters for the considered taxon). Our analysis of the score distributions (Figure 10) clearly shows that this prior estimation provides an efficient discrimination between promoters of orthologs and random selections. In addition, our comparison between programs suggests that the MAP is generally not the best score, since the total information content (which is also based on prior probabilities) shows better performances on the ROC curves (Figure 10C,F).

#### Choice of the motif width

An important advantage of *dyad-analysis *is that it does not require prior assumptions about motif width. Indeed, different widths are automatically detected since the program scans all spacing values between user-specified limits (from 0 to 20 in our case). By contrast, matrix-based approaches generally require for the user to specify a matrix width. An exception is the programs MEME, which permits to specify a range of matrix widths, and returns the motifs with the lowest E-value.

#### Expected number of sites

Another important parameter required by most matrix-based pattern discovery programs is an estimate of the expected number of sites (instances of the motif in the input sequence set). This parameter is very hard to estimate, since it depends on the transcription factor itself: some factors have 2 or 3 binding sites per promoter, whereas others have a single binding site. With enumerative algorithms like *oligo-analysis *and *dyad-analysis*, there is no need to provide prior estimates of the number of transcription-factor binding sites. Rather, the number of sites can be deduced *a posteriori*, by inspecting the feature maps of discovered words/dyads.

#### Negative control

An essential quality of a pattern discovery program is its capability to return a negative answer when, for some reason, the input sequences do not share any regulatory motif. In many articles, this aspect is simply neglected, and the evaluation is restricted to an analysis of promoters of co-regulated genes. In other cases, the negative control is performed, but it is based on a set of artificial sequences is generated by shuffling the original sequences [52]. Such artificial sequences already provide some level of control, but a more stringent test is to submit a random selection of real bacterial promoters [54]. Each of these promoters is likely to contain some binding sites for some transcription factors, but in principle no specific motif should be over-represented in random selections. Beyond its stringency, this negative control is also more realistic, because it mimics the situation where a biologists tries to detect motifs in bad clusters of genes. We thus chose to base our negative control on random selections of bacterial promoters.

#### Treatment of redundancy

The first implementation of the *gibbs *sampler [2] was accompanied by a program called *purge*, designed to discard redundant sequences. Indeed, the inclusion of multiple closely related sequences provokes a systematic repetition of all their fragments, which prevent the sampler from identifying the correct motifs. The effect of redundant sequences is very strong for footprint detection, because some small taxonomic groups are over-represented in genome databases. For example, the current NCBI collection (July 2007) contains 38 species for the family of Enterobacteriaceae, among which 8 strains of *Escherichia coli*. An approach to circumvent this problem has been to manually select a set of non-redundant genomes [52,19]. We address the problem in a more automated way, by applying a gene-wise sequence purging: for each set of orthologs, redundant sequences are masked before the pattern discovery step, but in a second time pattern matching is applied on the full set of non-purged sequences, in order to visualize motif conservation across all available species.

#### Site positions

Previous analysis of the upstream distribution of cis-regulatory elements in conserved groups of genes shows that positions of TFBS are conserved across species [59]. Consistently, the feature maps show a high conservation of the positions of putative LexA binding sites in Cyanobacteria (Figure 7C), Firmicutes (Figure 7D), Beta-proteobacteria (Figure 7F), Gamma-proteobacteria (Figure 7H) and Xanthomonadales (Figure 8), respectively. By contrast, position of the predicted sites are less conserved for two other taxonomical classes (Actinobacteria and Alpha-proteobacteria). This might either reflect a more heterogeneous collection of available genomes in this taxon, or that a more rapid turn-over of TF binding sites. A more detailed gene-per-gene study of motif locations within this group would be required to clarify this question.

## Conclusion

As pointed out by McCue and co-workers, many parameters can influence the results of phylogenetic footprinting, such as the number of sequences and the selected organisms [52]. We extended this question by assessing the impact of various parameters on the significance and correctness of discovered motifs in 368 genes. We tested all possible combinations between the following parameters: 2 background models, 5 taxonomical levels, operon inference, organism-specific dyad filtering, significance threshold. This quantitative evaluation suggests that the global optimum is generally found at intermediate taxonomical levels, and the class (Gamma-proteobacteria) seems to ensure the best tradeoff between sensitivity and specificity. However, beyond this general trend, the optimal level varies from gene to gene. For the detection of motifs in genes for which no regulation is known, we thus recommend to apply pattern discovery at all the taxonomical levels, and to inspect the feature maps in order to analyze the distribution of the predicted sites across the promoters of each taxon.

Furthermore, our evaluation shows that phylogenetic footprint detection is significantly improved by choosing a taxon-wide background model and by applying organism-specific dyad filtering.

In the second part of the article, we showed how a cross-taxonomical exploration of phylogenetic footprints can reveal the conservation and divergence of cis-regulation. This strategy was illustrated with the example of LexA auto-regulation, for which our predictions are remarkably consistent with the binding sites characterized in different taxonomical groups. Our method also detects a significant motif in Beta-proteobacteria, which has not yet been experimentally characterized. The predicted Beta-proteobacterial motif is similar to the one known in Gamma-proteobacteria, suggesting that cis-regulation has been conserved across these two groups. In addition, we highlight a partial divergence between the motifs detected in two subclasses of Gram-positive bacteria (Actinobacteria and Firmicutes), suggesting a progressive evolution of LexA binding specificity. These features illustrate the predictive power of the method, and its capability to track the evolutionary divergence of cis-regulatory motifs.

In summary, this assessment can be considered as a calibration, which should help biologists to estimate the relevance of each predicted motif, and guide their experimental work by producing testable hypothesis about the regulation of genes with no annotated function.

In the future, this approach opens the way to identify groups of co-regulated genes (regulons), by regrouping genes with similar motifs, in order to address the challenging domain of the evolution of transcriptional regulatory networks.

## Methods

### Prokaryotes genomes and orthologs selection

Fully sequenced prokaryote genomes were downloaded from NCBI [60] and installed in the Regulatory Sequence Analysis Tools [61]. Pairwise gene similarities were detected using BLAST [62] between the translated sequence of all the genes from *E. coli K12 *and 286 other bacterial genomes (12 September 2005) (see supplementary material). Orthologs were defined as bi-directional best hits (BBH) [63] with an E-value smaller than 10^-5^. The program *get-orthologs*, has been developed in order to select hits according to user-specified criteria [64].

### Pattern discovery in the taxonomic tree

We used Regulatory Sequence Analysis Tools [61,65,30] for sequence retrieval (*retrieve-seq*), pattern discovery (*dyad-analysis*), pattern matching (*dna-pattern*), feature map drawing (*feature-map*) and pattern comparison (*compare-patterns*).

In order to manage these tasks at each level of a taxonomic tree, we implemented in Perl a program called *footprint-analysis*. The program takes as input a taxonomic tree and a set of input sequences. We extracted the prokaryotic tree from the NCBI taxonomy database [66,67]. Starting from a set of input genes and their orthologous clusters, *footprint-analysis *retrieves the corresponding upstream sequences up to the upstream neighbor gene, with a maximal length of 400 bp, purges the sequences to discard large redundant fragments (>= 30 bp identical), and runs *dyad-analysis *at each taxonomical level, with the following options: no self-overlap between word occurrences (*-noov*), lower threshold of significance of 0 (*-lth occ_sig 0*), spacing comprised between 0 and 20 nucleotides (*-sp 0–20*). The analysis is restricted to groups containing at least three sequences.

### Prediction of operons

We predicted operons on the basis of a simple distance-based method inspired from Salgado and co-workers [40]. Rather than calculating a training-based log-likelihood, we classify an intergenic region as *within-operon *(*WO*) if it has a length smaller than 55 bp, and as *transcription unit border *(*TUB*) otherwise. We evaluated this method on 407 annotated operons from *E. coli K12 *[43] and 351 operons from *Bacillus subtilis *[68,69], and observed that, despite its simplicity, this distance-based threshold strategy gives a comparable accuracy (~80%) to that obtained with more elaborated methods.

### Sequence purging

An essential precaution for pattern discovery is to avoid statistical biases due to sequence redundancy. This problem is classically addressed by purging sequences, i.e. discarding repeated fragments. Sequence purging is even more important for promoters of orthologous genes, because the collection of sequenced genomes contains several closely related strains (for example, there are currently 5 strains of the species *E. coli*). If the selected species are too close from each other, the whole promoter sequence will be conserved, which will lead to an over-estimation of the significance for all the dyads found in these regions. We used the programs *mkvtree *and *vmatch *[70,71] to mask redundant fragments larger than 30 bp.

### Background models

The program *dyad-analysis *lets the user specify a background model to estimate prior dyad frequencies for the calculation of the significance score [9]. Two alternative background models have been tested in this study. A first possibility is to estimate the prior probability of each dyad as the product of the frequencies observed in the input sequences for its two spaced words (monads).

*P*(*W*_1_*n*_*x*_*W*_2_) = *F*(*W*_1_)*F(*W*_2_).

where *W1 *and *W2 *are the two monads (in our case, trinucleotides) forming the dyad, *n*_*x *_represents the spacing of length *x *between these trinucleotides, and *F*(*W*_*i*_) is the frequency of the word *W*_*i *_in the input sequence set. This model is referred to as MONAD in the rest of the text.

In the second background model (TAXFREQ), the prior probability of each dyad is estimated by computing the frequency of the same dyad in a taxon-specific reference set (sequences upstream of all genes of all organisms belonging to the considered taxon).

### Organism-specific dyads filtering

The search of phylogenetic footprints is generally motivated by the interest in one particular "reference" organism (e.g. *E. coli*). However, when patterns are discovered in promoters of a set of orthologous genes, some motifs can be significant due to their presence in other orthologs, whilst being absent from the promoter of the reference organism. Such motifs can indeed be involved in the regulation of some subgroups of the considered taxon, but not for the reference organism. In order to filter out such motifs, we apply an organism-specific filter, by discarding all the dyads that are not found in the promoter of the reference organism (*E. coli *in our case). Since the number of tested dyads is drastically lowered, the multi-testing correction is lower than with the full analysis. Consequently, for the same P-value, one generally has a lower E-value, and thus a higher significance. This organism-specific dyad filtering has thus a double effect of lowering the rate of false positives (by filtering our irrelevant dyads) and increasing the sensitivity (by increasing the significance of the relevant dyads).

### Heat map

The significance heat map (patterns versus taxa) was drawn with the statistical freeware R[72], using the function *heatmap.2() *from the package gplots. Similarities *S*_*ij *_were calculated between each pair *i*, *j *of significant patterns with the program *compare-patterns *(RSAT). The similarity matrix *S *was then converted to a distance matrix (*D*_*ij *_= *max*(*S*)-*S*_*ij*_), used to cluster patterns using a hierarchical clustering algorithm *hclust() *with the single linkage method. In order to simplify the visualization, we selected only patterns having a score at least equal to 8 in at least one taxon. Heat maps with other thresholds are available in the Additional file 1.

### Annotated motifs

In order to validate our analysis, the discovered motifs (significant dyads) were compared to annotated sites. For the analysis of LexA auto-regulation in all taxa, taxon-specific motifs were collected from the biological literature (Figure 2). For our systemic validation, we used annotated sites for 368 genes (coding sequences) from *E. coli K12 *annotated in RegulonDB [33] (updated in 11/2005). From 402 genes having annotated sites in the original set, we discarded 31 genes corresponding to non translated RNA (tRNA, sRNA, rRNA) and 3 genes without identified orthologs (2 leader peptides: *trpL *and *ilvL*; and *asr*, an heat shock polypeptide). The exact position of binding sites is not always perfectly identified, due to experimental imprecision. For this reason, we preferred to maintain the site-flanking sequences for this evaluation (10 bp on each side).

### Comparison between discovered motifs and annotated sites

The program *compare-patterns*, takes as input two collections of patterns (in this case, a set of discovered dyads and a set of annotated binding sites), and calculates the number of matching bases between each pair of patterns, as well as the statistical significance of the matches. We consider only perfect matches.

We define the following statistics:

- *Matching dyad (MD): *discovered dyad matching at least one annotated site for the considered gene.

- *Non-matching dyad (ND): *discovered dyads without any match with the annotated sites for the considered gene.

- *Matched site (MS): *annotated site matched by at least one discovered dyad.

- *Non-matched site (NS): *annotated site without any match with the discovered dyads.

- *Matched factor (MF): *transcription factor for which at least one of the binding sites has been matched by at least one of the discovered dyads in at least one of the target genes.

- *Non-matched factor (NF): *transcription factors for which none of the binding sites has been matched by any of the discovered dyads in any of the target genes.

#### Matching statistics

From the pattern comparison results, we derive the following evaluation parameters:

(1) The *positive predictive value (PPV) *is the fraction of discovered patterns matching at least one annotated site. *PPV *= *MD*/(*MD+ND*)

(2) *The sensitivity (Sn) *is the fraction of annotated binding sites matched by at least one discovered pattern. *Sn *= *MS*/(*MS+NS*)

(3) The tradeoff between sensitivity (*Sn*) and selectivity (as estimated by the *PPV*) can be captured by the geometric accuracy (*Acc.g*), defined as the geometric average of the sensitivity and PPV. $Acc\text{.}g=\sqrt{Sn\cdot PPV}$. The geometric accuracy is more severe than the frequently used arithmetic mean, because it puts a higher penalty on unbalanced predictions (e.g. low Sn with high PPV, or low PPV with high Sn).

(4) The *transcription factor-wise sensitivity (tf.Sn) *is the fraction of transcription factors for which at least one of the binding sites has been matched by at least one of the discovered dyads in one of the target genes. *tf.Sn *= *MF*/(*MF+NF*)

### Random promoter selections

We used the program *random-seq-select *(RSAT) to select, at each taxonomical level, 100 random sets of promoters. For each taxonomical level, the number of sequences per random set was defined as the average number of sequences obtained by group of orthologous genes (80 sequences per set for Bacteria, 49 for Proteobacteria, 32 for Gamma-proteobacteria, 16 for Enterobacteriales and 4 for Escherichia).

### Matrix-based pattern discovery

We used the following programs for matrix-based pattern discovery: MEME [57], gibbs [4], AlignACE [54], MotifSampler [11]. MEME relies on an expectation-maximization algorithm, whereas gibbs, AlignACE and MotifSampler rely on a Gibbs sampling algorithm, each implementation presenting some interesting characteristics.

#### Matrix width

the motif width (number of columns of the matrices) was set to 16 for all programs, except for MEME, which includes an option for testing all matrix width within a given range (we tested from 6 to 25).

#### Number of motifs

MEME, gibbs and MotifSampler support the detection of multiple motifs. We set the number of motifs per sequences to 5, and selected the top-ranking motif for each program. AlignACE detects a single motif per analysis.

The other program-specific options are detailed on the supporting web site.

The program *convert-matrix *of RSAT was used to convert the output files of these matrix-based programs into a homogeneous format. The conversion also includes the computation of total information content (TIC) and information per column (IPC), following Hertz and Stormo's formula for information content [8]:

$$\begin{array}{ccc} TIC=\sum_{j=1}^{w}\sum_{i=1}^{A}f_{i,j}^{'}\ln\left(\frac{f_{i,j}^{'}}{p_{i}}\right) & f_{i,j}^{'}=\frac{n_{i,j}+p_{i}k}{\sum_{i=1}^{A}n_{i,j}+k} & IPC=\frac{TIC}{w} \end{array}$$

where *f*_*i*,*j *_is the frequency of *i*^*th *^residue (matrix row) in the *j*^*th *^position of the motif (column of the matrix); *A *is the size of the alphabet (A = 4 for DNA), w is the matrix width, *p*_*i *_is the prior residue of the *i*^*th *^residue, and *k *is a pseudo-weight (set to 1 by default).

## Availability and requirements

Project name: Regulatory Sequence Analysis Tools (RSAT)

Project home page: 

Operating system(s): The main access to Regulatory Sequence Analysis Tools is via the web interface [61], which is platform independent. The Regulatory Sequence Analysis Tools can be used as stand-alone application on Unix-based systems (tested on Linux, Mac OSX, SPARC Solaris, Alpha Dec);

Programming language: Perl.

Licence: The web site is freely available to all users. The stand-alone version is freely available for academic users. Commercial companies are allowed to use the site during a reasonable testing period.

Any restrictions to use: non-commercial, non-military and non-redistribution.

Supplementary methods, scripts and predictions are available on the RSAT supplementary material [73].

## Abbreviations

Sig: Significance score; MD: Matching dyads; ND: Non-matching dyad; MS: Matched site; NS: Non-matched site; MF: Matched factor; NF: Non-matched factor: FP: False Positive; TP: True Positive; Sn: Sensitivity; PPV: Positive Predictive Value; Acc.g: geometric Accuracy; Sn.tf: Transcription Factor-wise Sensitivity; TIC: total information content; MAP: maximum a posteriori probability; TF: transcription factor; TFBS: transcription factor binding site.

## Authors' contributions

RJ collected the data, implemented the pipeline of RSAT programs, made the analysis and ran the evaluation procedures. JvH conceived the study and participated in its design, coordination, and in defining the matching statistics. Both authors were equally involved in writing the manuscript.

## Supplementary Material

Additional file 1 Correctness of dyads predicted by group of genes and taxonomical level, with all combinations of parameters. See Figure 3 for legend. Click here for file

Additional file 2 Correctness of dyads predicted by group of genes and taxonomical level, at different significance thresholds. Each heat map is drawn for a given threshold on the significance score. This threshold increases from left to right: 0, 1, 1.5, 2, 3, 4 and 5. We used here the results of dyad-analysis using the MONAD background model, the dyads filtering and the operons prediction. See Figure 3 for legend. Click here for file

Additional file 3 Significance heat map of the dyads discovered at different taxonomical levels in upstream sequences of lexA orthologs (See figure 6 for legend). Click here for file
