# Supplementary material for: Evaluation of phylogenetic footprint discovery for predicting bacterial cis-regulatory elements and revealing their evolution
Source: BMC Bioinformatics. 2008 Jan 23;9:37. doi: 10.1186/1471-2105-9-37 (PMC2248561; doi:10.1186/1471-2105-9-37)
Supplement: Additional file 1 — Correctness of dyads predicted by group of genes and taxonomical level, with all combinations of parameters. See Figure 3 for legend. [file 1471-2105-9-37-S1.ppt]

## Slide 1
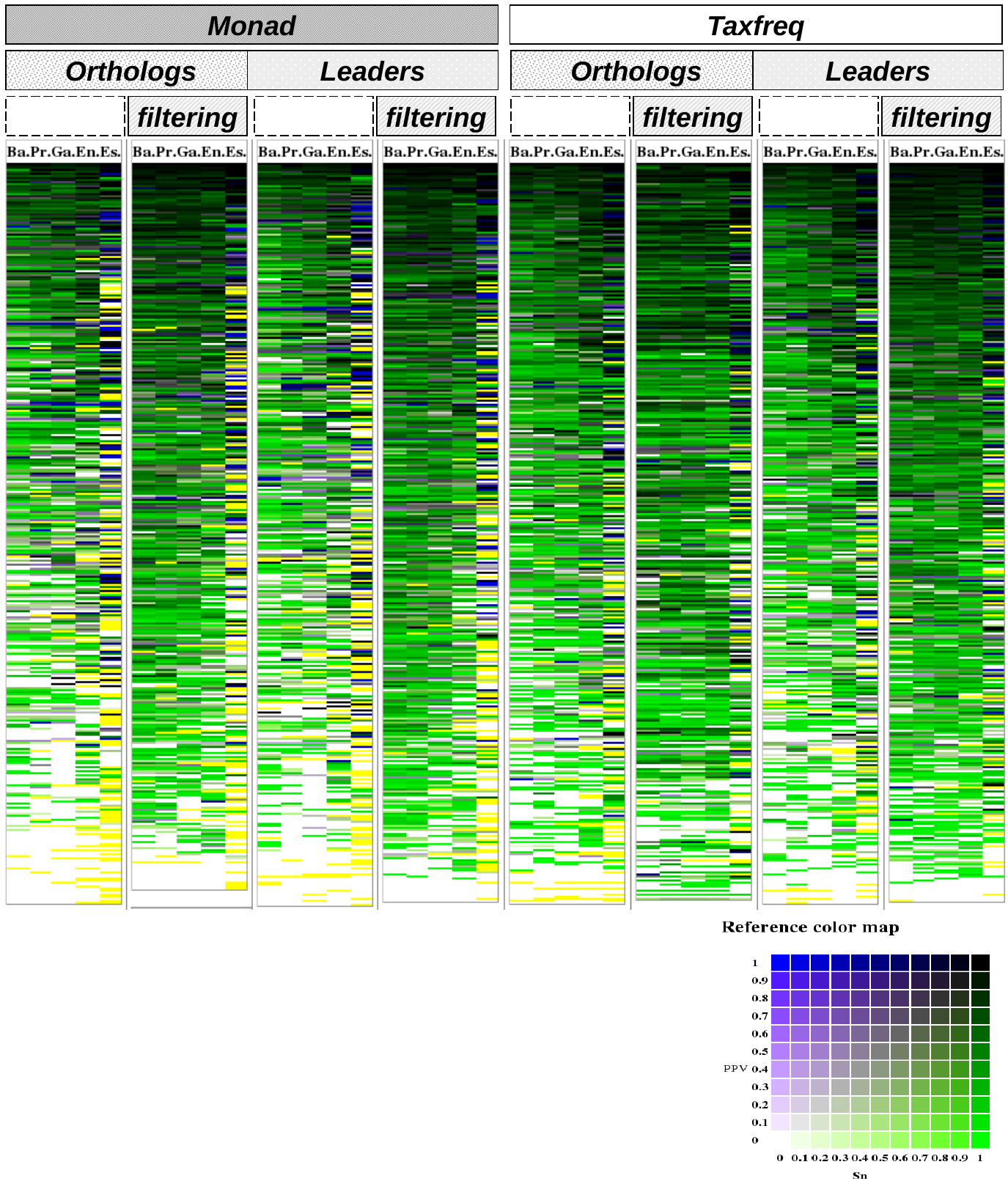

Monad
Taxfreq
Orthologs
Leaders
Orthologs
Leaders
filtering
filtering
filtering
filtering
