# Supplementary figures and images for: Evaluation of phylogenetic footprint discovery for predicting bacterial cis-regulatory elements and revealing their evolution
Source: BMC Bioinformatics. 2008 Jan 23;9:37. doi: 10.1186/1471-2105-9-37 (PMC2248561; doi:10.1186/1471-2105-9-37)

## Slide 1
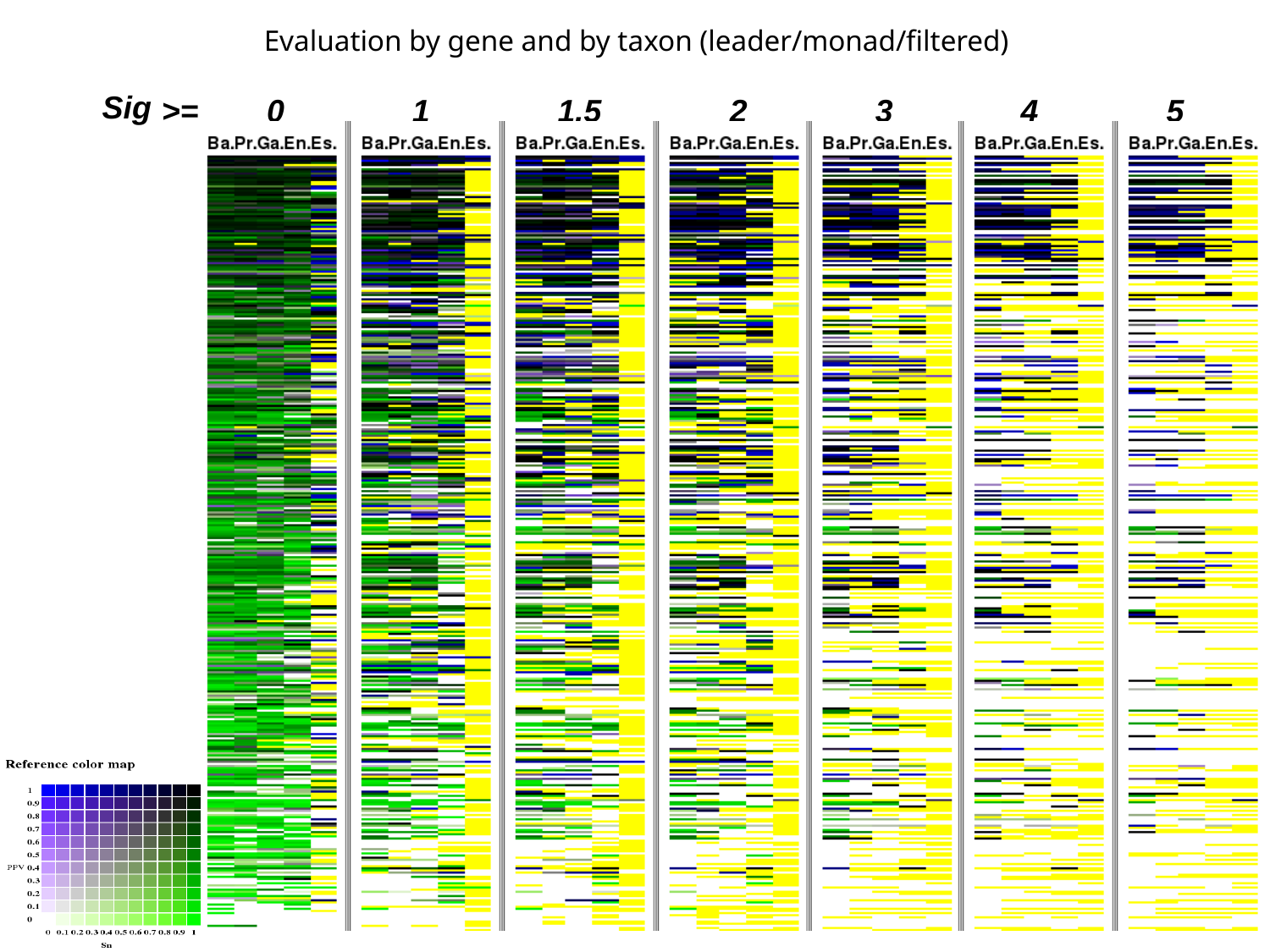

Evaluation by gene and by taxon (leader/monad/filtered)
Sig
>=
0
1
1.5
2
3
4
5

Supplement: Additional file 2 — Correctness of dyads predicted by group of genes and taxonomical level, at different significance thresholds. Each heat map is drawn for a given threshold on the significance score. This threshold increases from left to right: 0, 1, 1.5, 2, 3, 4 and 5. We used here the results of dyad-analysis using the MONAD background model, the dyads filtering and the operons prediction. See Figure 3 for legend. [file 1471-2105-9-37-S2.ppt]

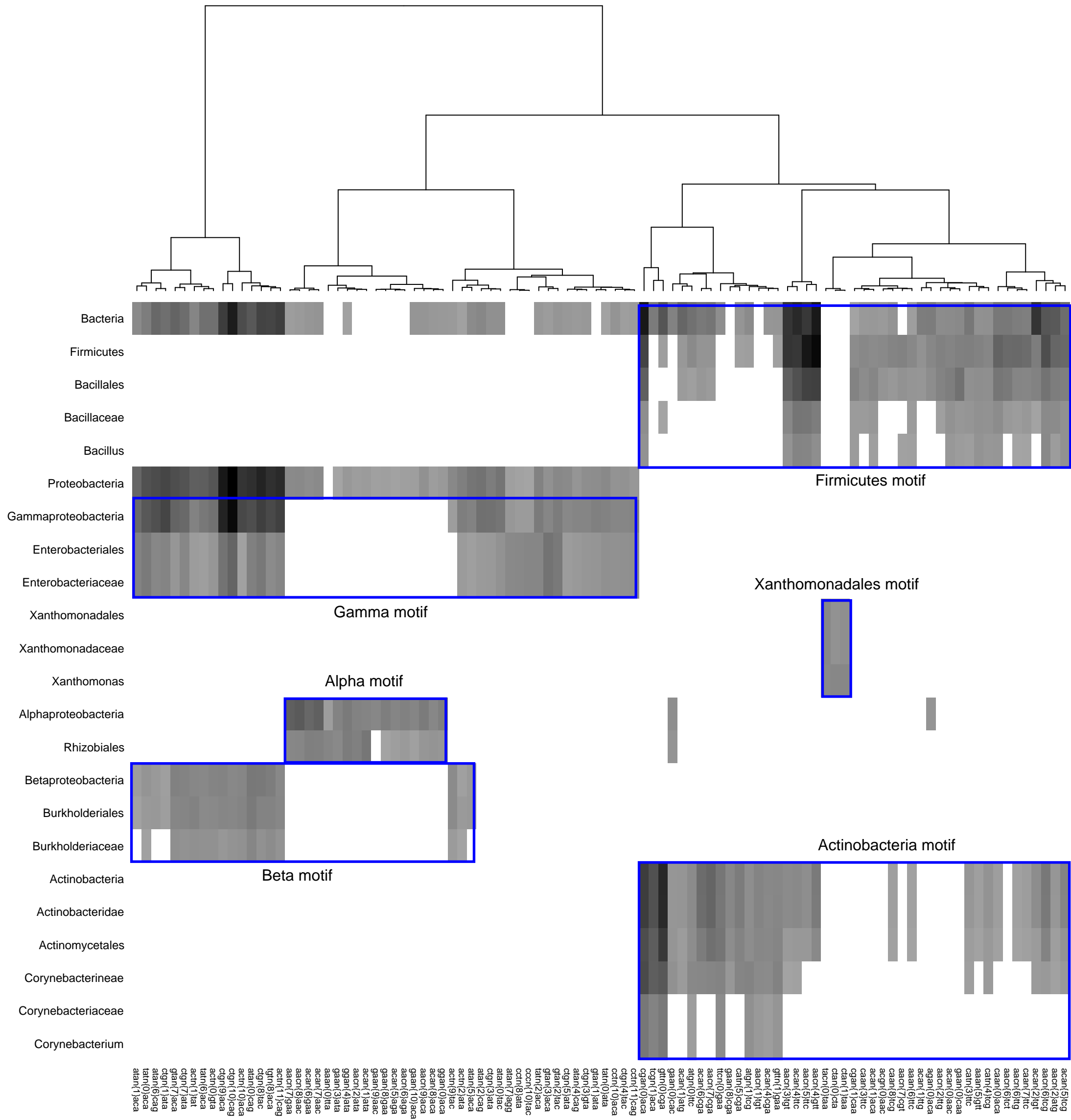

Supplement: Additional file 3 — Significance heat map of the dyads discovered at different taxonomical levels in upstream sequences of lexA orthologs (See figure 6 for legend). [file 1471-2105-9-37-S3.pdf]
